# Joint non-uniform sampling of all incremented time delays for quicker acquisition in protein relaxation studies

**DOI:** 10.1007/s10858-017-0115-8

**Published:** 2017-05-15

**Authors:** Mateusz Urbańczyk, Michał Nowakowski, Wiktor Koźmiński, Krzysztof Kazimierczuk

**Affiliations:** 1 Centre of New Technologies, University of Warsaw, Banacha 2C, 02-097 Warsaw, Poland; 2 Spektrino Sp. z o.o., Żwirki i Wigury 89, 02-089 Warsaw, Poland; 3 Faculty of Chemistry, Biological and Chemical Research Centre, University of Warsaw, Żwirki i Wigury 101, 02-089 Warsaw, Poland

**Keywords:** Relaxometry, Non-uniform sampling, Compressed sensing, Fast NMR, Protein dynamics

## Abstract

NMR relaxometry plays crucial role in studies of protein dynamics. The measurement of longitudinal and transverse relaxation rates of $^{15}$N is the main source of information on backbone motions. However, even the most basic approach exploiting a series of $^{15}$N HSQC spectra can require several hours of measurement time. Standard non-uniform sampling (NUS), i.e. random under-sampling of indirect time domain, typically cannot reduce this by more than 2–4$\times$ due to relatively low “compressibility” of these spectra. In this paper we propose an extension of NUS to relaxation delays. The two-dimensional space of $t_1$/$t_{relax}$ is sampled in a way similar to NUS of $t_1$/$t_2$ domain in 3D spectra. The signal is also processed in a way similar to that known from 3D NUS spectra i.e. using one of the most popular compressed sensing algorithms, iterative soft thresholding. The 2D Fourier transform matrix is replaced with mixed inverse Laplace-Fourier transform matrix. The peak positions in resulting 3D spectrum are characterized by two frequency coordinates and relaxation rate and thus no additional fitting of exponential curves is required. The method is tested on three globular proteins, providing satisfactory results in a time corresponding to acquisition of two conventional $^{15}$N HSQC spectra.

## Introduction

Dynamics of protein molecules is typically studied by heteronuclear NMR relaxation measurements (Key et al. 1989). A number of techniques based on $^{15}$N, $^{13}$C and $^{2}$H resonance provides an insight into motions on time-scales ranging from picoseconds to hours (Palmer 2004) allowing to characterize catalysis (Shapiro et al. 2000; Eisenmesser et al. 2005), binding (Akke et al. 1993; Hodsdon and Cistola 1997), folding (Prompers and Brüschweiler 2002; Neudecker et al. 2007) and other phenomena. Usually, a set of $^{15}$N- or $^{13}$C-edited two-dimensional spectra is acquired to probe the relaxation of a particular coherence under different conditions, e.g. for various durations of a relaxation delay in a pulse sequence. The relaxation rate is then extracted from peak intensities (Fushman 2008) which thus have to be measured with a high fidelity. Typically, a curve corresponding to a given model is fit to a peak intensity plotted as a function of relaxation delay.

Of course peak overlap, common in 2D spectra of even small proteins, complicates the analysis of relaxation data (Koskela et al. 2004). It is important to note, that while extensive sampling of time dimension improves a frequency resolution, probing more values of a relaxation delay provides better determination of decay rates. Typically one needs at least 128 points in $t_1$ (for typical $^{15}$N HSQC experiment with spectra width of about 2.5 kHz) and several lengths of relaxation delay which results in at least 3–4 hours long experiment. As shown e.g. by Stetz and Wand (2016), every 2D spectrum in a series can be accelerated by non-uniform sampling (NUS), and reconstructed by one of the methods reported e.g. compressed sensing (CS) (Kazimierczuk and Orekhov 2011; Holland et al. 2011), maximum entropy (Laue et al. 1986; Mobli and Hoch 2008) or SIFT (Matsuki et al. 2009). However, since the quality of NUS reconstruction depends mostly on the absolute number of points compared with the number of peaks in a spectrum (Shchukina et al. 2016) achievable gains on time are not significant. Simply, typical 2D $^{15}$N HSQC spectrum of a protein is not sparse enough to go below 50–70 sampling points. The efficient alternative is to apply a multidimensional decomposition method treating a series of 2D NUS spectra as one 3D object, which greatly improves the reconstruction comparing to separate CS processing of each individual 2D dataset (Linnet and Teilum 2016).

In this paper, we will show, how CS method applied usually to non-uniformly sampled oscillating signals can be extended to process the data that includes “relaxation dimensions”. Similarly to co-processing using multidimensional decomposition (coMDD), the method also treats a series of 2D datasets as one 3D object, but (as all CS algorithms) exploits the fact, that such an object has a sparse representation in certain domain. It has two advantages over 3D coMDD processing discussed recently in Linnet and Teilum (2016). Firstly, it allows joint NUS of both domains, which means, that extensive sampling of a relaxation decay is possible, even for low sampling levels. Secondly, it provides relaxation rates at the output since “relaxation dimension” is processed with inverse Laplace transform [as proposed before for full sampling (Koskela et al. 2004)]. Recently, we have shown the application of the method to the diffusion-ordered spectroscopy (DOSY), where 3D HSQC-iDOSY experiment was run in a time comparable to two conventional 2D HSQC measurements (Urbańczyk et al. 2014). Here, we show that similarly fast and accurate results can be obtained in the case of protein relaxation experiments.

## Methods

In this section we describe the experimental setup and processing details of $^{15}$N-T$_1$-HSQC experiment that was used to test joint FT-ILT procedure proposed in this study.

### Joint FT-ILT

The time-domain signal in two-dimensional experiment with $t_1$ (indirect) and $t_2$ (direct) time dimensions can be described as:

1 $$s\left( t_1,t_2 \right) =\sum _{i=1}^{N}s_i(t_1)\otimes s_i(t_2)$$

where *N* is a number of pairs of nuclei contributing to the signal. The spectrum ${\mathbf{S}}$ is obtained from FID vector ${\mathbf {s}}$ as a solution to the following system of equations:

2 $$\mathbf {F}\mathbf {S}=\mathbf {s}$$

where ${\mathbf {F}}$ stands for the inverse Fourier transform. The system of equations is unique if ${\mathbf {s}}$ and ${\mathbf {S}}$ are of equal size. In case of sparse non-uniform sampling ${\mathbf {s}}$ is shorter than ${\mathbf {S}}.$ The problem can be solved by sparsity-constrained CS approach, where ${\mathbf {S}}$ is found by minimizing the functional involving the sparsity-enforcing term $\Vert {\mathbf {S}} \Vert _{\ell _1}$ (Candès and Wakin 2008):

3 $$\min_{\mathbf {S}} \Vert \mathbf {F} \mathbf {S} -\mathbf {s} \Vert _{\ell _2}^{2}+\tau \Vert {\mathbf {S}} \Vert _{\ell _1}$$

where $\tau$ is Lagrange coefficient keeping the balance between the two terms. The method has been successfully employed in NMR (Kazimierczuk and Orekhov 2011; Holland et al. 2011) and improved by many other groups (Hyberts et al. 2012; Sun et al. 2015).

The signal can be extended by adding a relaxation “pseudo-dimension”, i.e. by repeating the acquisition for various settings of relaxation delay in the pulse sequence:

4 $$s(t_1,t_2,t_{relax})=\sum _{i=1}^{N} s_i(t_1)\otimes s_i(t_2)\otimes \alpha _i(t_{relax})$$

where $\alpha _i(t_{relax})$ is an exponentially decaying function of a relaxation delay. Traditionally, dimensions of such a signal are processed sequentially. Firstly, time dimensions are processed with Fourier transform (or with NUS reconstruction algorithm, if undersampled). Then, exponential curve is fitted to the peak intensity versus relaxation delay plot to find the relaxation rate. Alternatively, especially for multi-exponential decays, it is convenient to use inverted Laplace transform to find decay rates:

5 $$\min _{\mathbf {A}} \Vert \mathbf {L} \mathbf {A} - {\alpha } \Vert _{\ell _2}^{2}+\tau \Vert {\mathbf {A}}\Vert _{\ell _1}$$

where ${\mathbf {L}}$ is Laplace transform matrix. This kind of regularization enforces the sparsity of the solution (Donoho 2006). Similar approach with maximum entropy regularization has been proposed for relaxation data by Koskela et al. (2004).

Recently, we have shown that $\ell _1$-norm regularization can be applied to all dimensions together (Urbańczyk et al. 2014).

The minimized function becomes:

6 $$\min _{\mathbf {Q}} \Vert {\mathbf {P}} {\mathbf {Q}} -{\mathbf {q}} \Vert _{\ell _2}^{2}+\tau \Vert {\mathbf {Q}}\Vert _{\ell _1}$$

where ${\mathbf {P}}$ is combined “Fourier-Laplace” transform:

7 $${\mathbf {P}}={\mathbf {F}}\otimes \mathbf {L}$$

and ${\mathbf {q}}$ is vector of non-uniformly sampled $t_1$/$t_{relax}$ part of 3D signal from Eq. 4, while ${\mathbf {Q}}$ is its spectrum. The minimization of six can be effectively performed using e.g. iterative soft thresholding algorithm (Stern et al. 2007). Our study employs a rapidly converging Fast Iterative Shrinkage thresholding algorithm (FISTA) variant of the method (Beck and Teboulle 2009).

### Experimental details

The method was tested on $^{15}$N-T$_1$-HSQC (Farrow et al. 1994) (the pulse sequence allowing joint NUS of $t_1$/$t_{relax}$ is available on request from authors) of three proteins (obtained from Giotto Biotech): SRC Homology(SH3) protein (1 mM protein sample in 10:90 $\text{D}_{2}\text{O}/\text{H}_{2}\text{O},$ 10 mM sodium citrate, 0,02% $\text{NaN}_3,$ pH 3.5), immunoglobulin binding domain of protein G (GB1, 1 mM protein sample in 10:90 $\text{D}_2\text{O}/\text{H}_2\text{O}$ 20 mM sodium phosphate and 0.02% $\text{NaN}_3,$ pH 7.0) and human ubiquitin (1 mM protein sample in 10:90 $\text{D}_{2}\text{O}/\text{H}_{2}\text{O},$ 50 mM phosphate buffer and 0.02% $\text{NaN}_3,$ pH 7.1). The experiments were carried out on Varian 700 MHz DDR2 spectrometer equipped with triple-resonance HCN probe. Measurements were performed at 298 K.

**Fig. 1:**
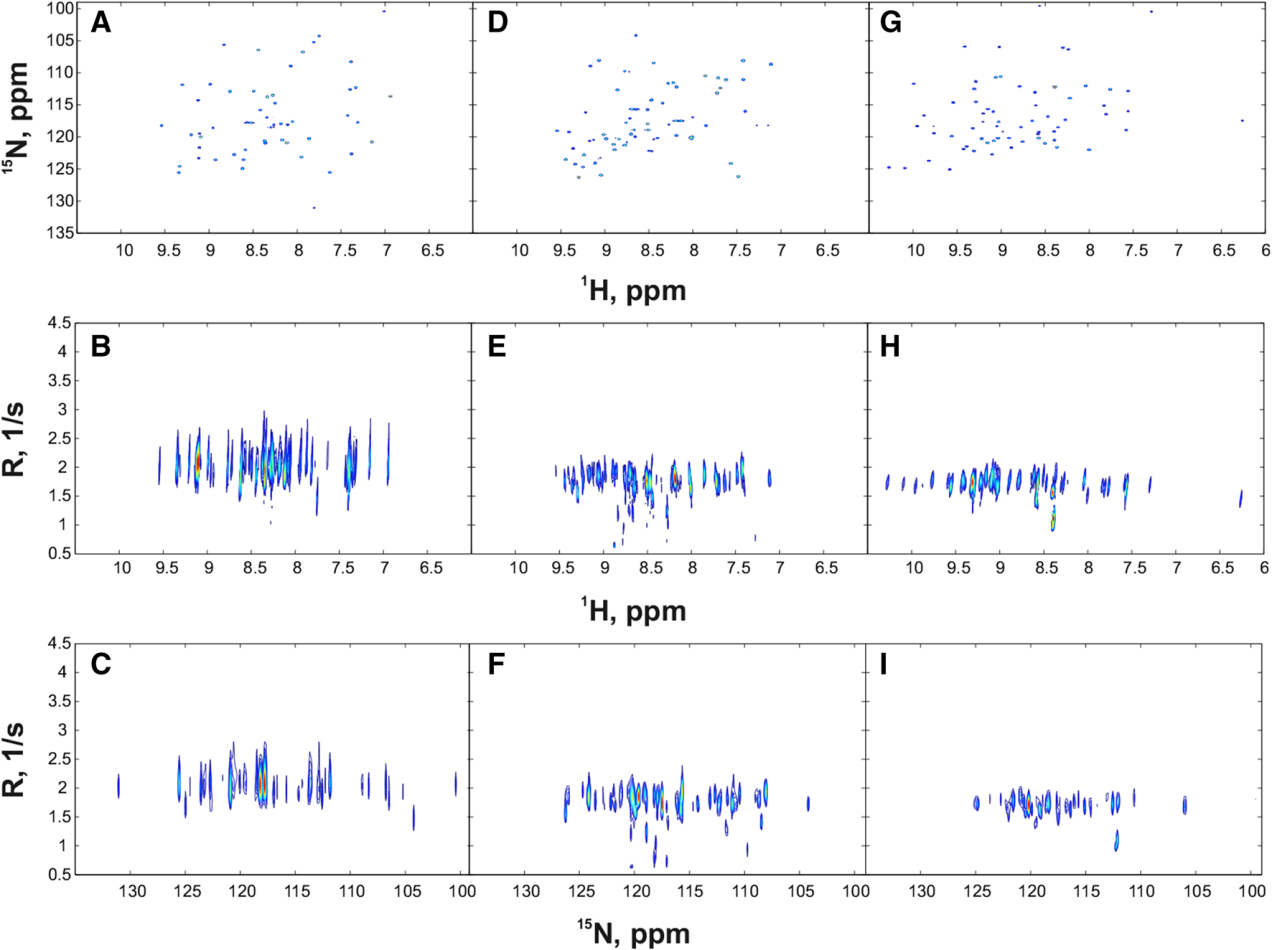
Projections of 3D T1-$^{15}$N-HSQC spectra of three protein samples. Projections of GB1 spectrum: **a** over relaxation dimension, **b** over $^{15}$N dimension, **c** over $^{1}$H dimension. Projections of SH3 spectrum: **d** over relaxation dimension, **e** over $^{15}$N dimension, **f** over $^{1}$H dimension. Projections of ubiquitin spectrum: **g** over relaxation dimension, **h** over $^{15}$N dimension, **i** over $^{1}$H dimension

For each protein, two experiments were carried out: conventional, with relaxation delay sampled with 10 points from 0.01 to 0.8 s; and joint NUS of $t_1$ and relaxation delay with 256 points (relaxation delay was randomized using a mesh size of 0.01 s). The former experiment was also used to create artificial NUS datasets for coMDD processing, as described in Linnet and Teilum (2016). The parameters were set to: number of iterations −50, MDD noise level −0.7, number of components −25. The latter was used to create datasets at varying sampling level i.e. 128, 144, 160, 176, 192, 208, 224, 240 and 256 points. In all cases, 2044 complex points were measured in the direct dimension with the spectral width of 12019.2 Hz. For the indirect ${^{15}\text{N}}$ dimension the grid of 128 points was used (fully sampled for classic and sparse sampled for the NUS experiment) with spectral width of 3000 Hz. The sampling schedules were generated using a web-tool written by the author. The tool is available at http://itamed.spektrino.com. All spectra were preprocessed in NMRPipe (Delaglio et al. 1995). The projections of 3D $^{15}$N-T$_1$-HSQC spectra sampled at 256 points are shown in the Fig. 1.

The conventionally sampled data was processed with FT and exponential curve fitting. Additionally, the full data was sub-sampled and processed with coMDD. Obtained full spectra were processed with exponential curve fitting to obtain decay rates.

The joint NUS experiments where processed using 2D ITAMeD program (Urbańczyk et al. 2014) with number of iterations set to 500 and $\lambda =100$ (both indirect dimensions processed together). The indirect *F*1 and relaxation dimensions in each spectrum were reconstructed using 256$\,\times \,$256 grid, with relaxation rate band set from 0.5 to 5 Hz (SH3 and ubiquitin) or from 0.1 to 9.5 Hz (GB1). To obtain the relaxation rates (*R*) from the datasets processed with ITAMeD, the peak-picking was performed by a MATLAB script that imported the frequency coordinates from the peak list manually prepared from the ${^1}$H–$^{15}$N projection and used Gaussian fit in the “relaxation” dimension. The top of a Gaussian peak determined the value of *R*.

## Results and discussion

**Fig. 2:**
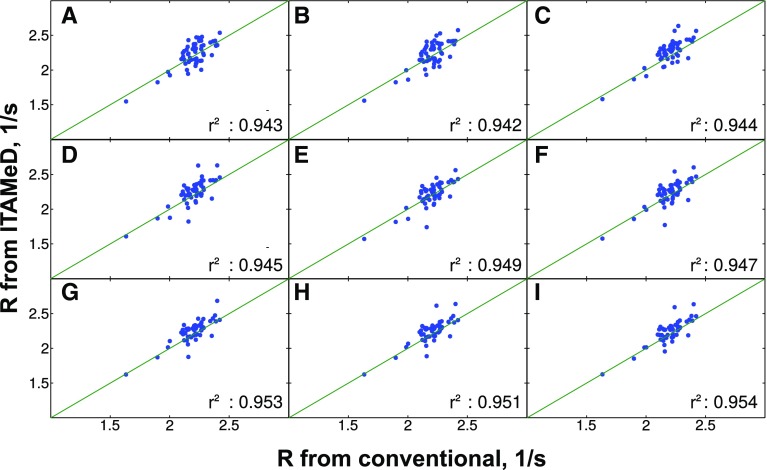
The comparison between longitudinal relaxation rates obtained with conventional method (mono-exponential fitting of peak intensities in a series of fully sampled spectra) and joint FT-ILT for sparse sampling at different sampling levels. The sample of immunoglobulin binding domain of protein G (GB1) was used. Each plot corresponds to different sampling levels for $t_1$/$t_{relax}$ domain: **a** 128 points, **b** 144 points, **c** 160 points, **d** 176 points, **e** 192 points, **f** 208 points, **g** 224 points, **h** 240 points, **i** 256 points. The correlation coefficient ($r^2$) between relaxation obtained from both methods is displayed

**Fig. 3:**
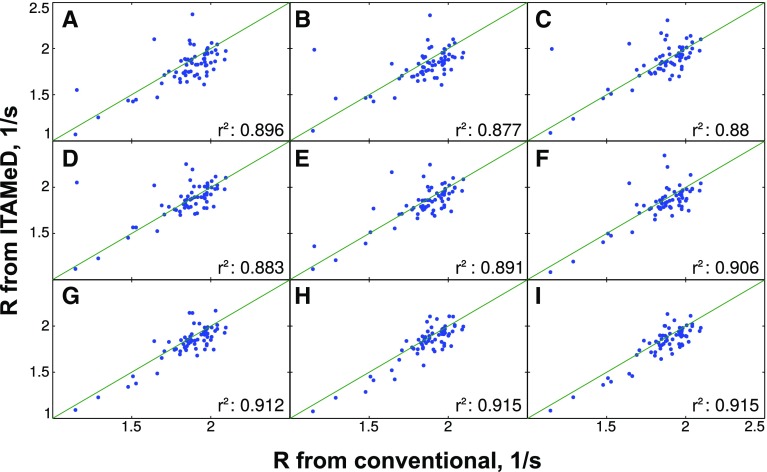
The comparison between longitudinal relaxation rates obtained with conventional method (mono-exponential fitting of peak intensities in a series of fully sampled spectra) and joint FT-ILT for sparse sampling at different sampling levels. The sample of SRC Homology (SH3) protein was used. *Each plot* corresponds to different sampling levels for $t_1$/$t_{relax}$ domain: **a** 128 points, **b** 144 points, **c** 160 points, **d** 176 points, **e** 192 points, **f** 208 points, **g** 224 points, **h** 240 points, **i** 256 points. Additionally the correlation between relaxation obtained from both methods is displayed ($r^2$)

**Fig. 4:**
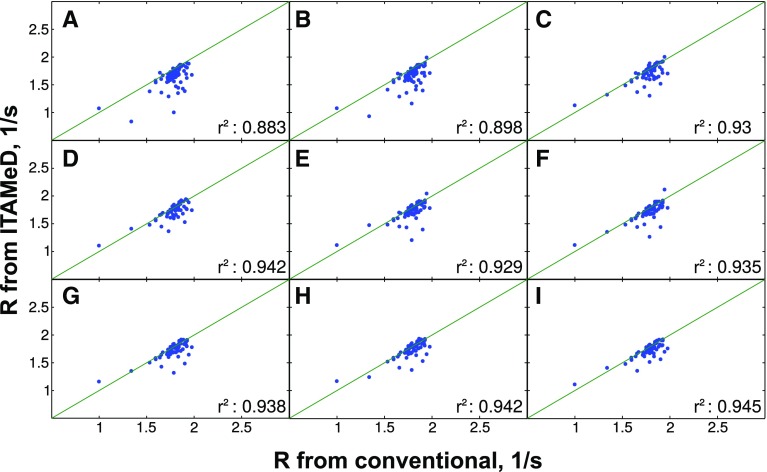
The comparison between longitudinal relaxation rates obtained with conventional method (mono-exponential fitting of peak intensities in a series of fully sampled spectra) and joint FT-ILT for sparse sampling at different sampling levels. The sample of human ubiquitin protein was used.* Each plot* corresponds to different sampling levels for $t_1$/$t_{relax}$ domain: **a** 128 points, **b** 144 points , **c**160 points, **d** 176 points, **e** 192 points, **f** 208 points, **g** 224 points, **h** 240 points, **i** 256 points. Additionally the correlation between relaxation obtained from both methods is displayed ($r^2$)

To check the dependence of reconstruction quality on the sampling level, NUS spectra were reconstructed from various numbers of NUS points from $t_1$/$t_{relax}$ space (128–256). Figures 2, 3 and 4 present the correlation of longitudinal relaxation rates *R* obtained from ITAMeD with the ones from conventional processing, i.e. Fourier transform followed by exponential fitting of peak intensities.

It is noteworthy, that conventional processing based on mono-exponential fitting does not provide a “perfect solution”. Although it is a good estimator of the decay rate for non-overlapping peaks, it may fail in several cases, as discussed in the literature: Tjandra et al. (1996), Koskela et al. (2004), Orekhov et al. (2004). The extensive comparisons of the results of joint FT/ILT have been done before on simulated data (Urbańczyk and Kazimierczuk 2013). The quality was also checked on experimental diffusion data (Urbańczyk et al. 2014) where “perfect solution” is also not provided, but coordinates in the diffusion dimension for different nuclei belonging to the same molecule have to match. Both studies have shown, that the ITAMeD estimates well the decay rates from even very small datasets.

The traditional exponential fitting of the relaxation curves provides, besides the decay rate, the error of the fit. The presented method could only provide combined “fit errors” for all $R_1$/*F*1 planes corresponding to a given spectral point of the direct dimension (which is not informative). Also, similarly as in traditional (frequency-domain) NUS spectra the infinite number of FIDs can fit the measured data exactly (zero error). Thus, peak widths in relaxation dimensions of the presented spectra should not be mistaken with an uncertainty of $R_1$, as they are also dependent on sparsity contraint $\lambda$.

**Fig. 5:**
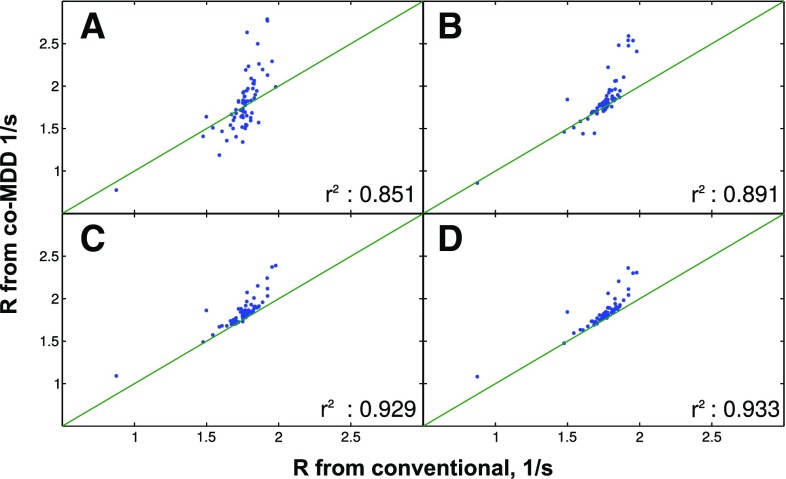
The comparison of conventional method (mono-exponential fitting of peak intensities in a series of fully sampled spectra) and coMDD processing of NUS data sampled at different sampling levels for a sample of human ubiquitin. **a**, **b** Subplot correspond to coMDD results of processing when all the planes are non-uniformly sampled (**a** 16 points per plane—160 total, **b** 32 points per plane—320 total). **c**, **d** Correspond to the results of coMDD processing with first plane fully sampled and the other nine planes NUS (**c** 16 points per plane—272 total, **d** 32 points per plane—416 total) additionally the correlation between relaxation obtained from both methods is displayed ($r^2$)

Similarly, the coMDD processing results for ubiquitin are shown in the Fig. 5. coMDD reconstruction was performed on four different datasets. First dataset consists of ten planes with 16 NUS points each, second dataset with 32 points and ten planes. The last two datasets consist of one fully sampled plane and nine NUS planes (16 points and 32 points each, accordingly). The processing exploiting one fully sampled spectrum is known to be more effective (Linnet and Teilum 2016), which was also confirmed in our study.

Interestingly, the ITAMeD results are stabilized at the level between 176 and 208 points. The coMDD method performs similarly well to ITAMeD only with the first spectrum fully sampled. Nevertheless, coMDD correlation for 416 sampled points in total is still worse than 176 points ITAMeD result. Of course, using limited numbers of sampling points has to be done with care when dealing with low concentration or other factors resulting in low sensitivity. On the other hand, for highly concentrated but unstable samples the sparse sampling may be the only reasonable choice.

Additionally, as reported before for diffusion studies (Urbańczyk et al. 2014) the noise is separated as false peaks at either very high or very low “relaxation rate”. Therefore the noise-related artifacts are easily recognizable, if the band of reconstructed decay rates is set wide enough.

We would like to emphasize, that the approach is the most effective in experiments where the exponential decay can be sampled off-grid or using very fine grid. The effectiveness would be reduced in the case of T$_2$ CPMG experiments, where sampling is restricted to an even number of echos (this limitation is less significant in the case of small molecules, when $T_2$ is relatively long). Finally, techniques where relaxation is not encoded as (multi)exponential decay (e.g. constant relaxation time CPMG dispersion experiments) are out of the scope of the method.

## Conclusions

We have shown, how to implement joint non-uniform sampling of indirect time dimension and relaxation delays in a series of 2D $^{15}$N HSQC spectra. The method can be easily extended to other relaxation experiments of any dimensionality. The relaxation pseudo-dimension is processed jointly with frequency dimensions by sparsity-regularized algorithm. Due to extensive sampling of a relaxation delay, the method provides good results in a short time.
